# Plasma 5-Fluorouracil Exposure, Clinical Outcomes, and Therapeutic Drug Monitoring in Advanced Colorectal Cancer

**DOI:** 10.3390/cancers18101673

**Published:** 2026-05-21

**Authors:** Naoki Sakuyama, Kiichi Nagayasu, Yu Abe, Takumi Ochiai, Futoshi Shibasaki

**Affiliations:** 1 Department of Surgery, The Institute of Medical Science, The University of Tokyo, Tokyo 108-8639, Japan; 2 Department of Surgery, Tokyo Metropolitan Tobu Chiiki Hospital, Tokyo 125-8512, Japan; kiichi_nagayasu@tmhp.jp (K.N.); hugh.abe.6.24@gmail.com (Y.A.); 3 Misato Care Center, Aiyukai Medical Corporation, Misato 341-0028, Saitama, Japan; takumi.ochiai@amg.or.jp; 4 Molecular Medical Research Project, Department of Genome Medicine, Tokyo Metropolitan Institute of Medical Science, 2-1-6 Kamikitazawa, Setagaya-ku, Tokyo 156-8506, Japan; shibasaki-f@tobira.tokyo

**Keywords:** 5-fluorouracil, therapeutic drug monitoring, pharmacokinetics, colorectal cancer, continuous infusion, toxicity, progression-free survival, overall survival, immunochromatography

## Abstract

Background/Objectives: Body-surface-area-based dosing of continuous-infusion 5-fluorouracil does not account for inter-individual pharmacokinetic variability. This pilot study explored whether patient-level representative plasma 5-fluorouracil exposure within the target area under the concentration–time curve (AUC) range was associated with clinical outcomes. It evaluated a prototype immunochromatographic assay as a preliminary monitoring tool. Methods: Fifteen patients with unresectable advanced or recurrent colorectal cancer who received continuous-infusion 5-fluorouracil-based chemotherapy were prospectively evaluated between 1 January 2017 and 30 April 2018. Plasma 5-fluorouracil levels were measured during eight treatment cycles at three time points in each cycle. Representative AUC values were calculated using median concentrations across cycles and interpreted as exploratory patient-level exposure indices. Tumor response, grade ≥2 adverse events, progression-free survival, and overall survival were assessed descriptively. Results: The median representative AUC was 24.3 mg·h/L. Eight patients (53.3%) were within the target range of 20–30 mg·h/L, whereas seven (46.7%) were outside it. Disease control was observed in 7 of 8 patients (87.5%) within the target range and in 3 of 7 patients (42.9%) outside it. Grade ≥2 adverse events were less frequent in the target-range group (2/8, 25.0%) than in the outside-range group (6/7, 85.7%; *p* = 0.041). Progression-free survival was numerically longer in the target-range group (17.2 vs. 9.2 months, *p* = 0.36), while overall survival did not differ clearly (*p* = 0.76); these survival analyses were exploratory. The prototype immunochromatographic assay showed a favorable correlation with the My-5FU assay (R2 = 0.762), but Bland–Altman analysis showed relatively wide limits of agreement. Conclusions: Target plasma 5-fluorouracil exposure was associated with lower clinically relevant toxicity and may support favorable tumor control in this pilot cohort. The prototype immunochromatographic method demonstrated preliminary feasibility for rapid plasma 5-FU monitoring but requires further validation before routine dose adjustment.

## Simple Summary

Continuous-infusion 5-fluorouracil remains a key component of chemotherapy for colorectal cancer, but plasma drug exposure varies considerably among patients even when the dose is determined according to body surface area. This pilot study explored whether patient-level representative 5-fluorouracil exposure within the target AUC range was associated with tumor control, toxicity, and exploratory survival outcomes. Patients within the target range showed a lower frequency of clinically relevant adverse events and numerically favorable tumor control, whereas survival analyses were descriptive and hypothesis-generating. We also performed a preliminary method-comparison analysis of a prototype immunochromatographic assay for plasma 5-fluorouracil measurement. Although the assay showed a favorable correlation with the My-5FU assay, Bland–Altman analysis demonstrated relatively wide limits of agreement. These findings support the feasibility of further developing simplified therapeutic drug monitoring tools. Still, larger prospective validation studies are required before this prototype assay can guide clinical dose adjustment.

## 1. Introduction

Clinical outcomes for patients with colorectal cancer (CRC) who undergo chemotherapy have improved substantially with advances in fluoropyrimidine-based combination regimens and the incorporation of molecularly targeted agents. Nevertheless, treatment response remains heterogeneous, and clinically significant adverse events continue to limit dose intensity and treatment continuity.

There is growing interest in individualized treatment strategies that account for inter-individual pharmacokinetic variability. The global burden of colorectal cancer remains substantial, with Japan experiencing some of the highest incidence rates in Asia, necessitating optimized treatment strategies for advanced cases [1,2,3]. While the shift toward precision oncology has predominantly focused on molecularly targeted biomarkers, optimizing cytotoxic mainstays such as 5-FU is equally vital for improving long-term survival [4,5,6,7]. Beyond the well-characterized DPD deficiency, inter-individual variability is also driven by genetic polymorphisms in enzymes such as thymidylate synthase (TYMS) and methylenetetrahydrofolate reductase (MTHFR), as well as by physiological factors such as renal function and nutritional status [4,5]. Sub-optimal systemic exposure is particularly concerning because “underexposure” not only results in poor tumor response but may also contribute to the selection of resistant tumor clones, leading to earlier disease progression [5,8,9].

In routine practice, the 5-fluorouracil (5-FU) dose is usually determined by body surface area (BSA), which incorporates height and body weight but does not account for metabolic or pharmacokinetic differences among patients. As a result, BSA-based dosing may lead to substantial variability in systemic 5-FU exposure, increasing the risk of underexposure, toxicity, or both [4,5].

Studies from Western countries have demonstrated major inter-individual differences in plasma 5-FU pharmacokinetics and have shown associations between systemic exposure, treatment response, and toxicity [8,9,10,11,12,13,14,15,16,17,18]. Japanese studies have also suggested that high plasma 5-FU concentrations are associated with an increased risk of adverse events, including severe hematologic toxicity [1]. In our previous preliminary study, we also reported the feasibility of measuring plasma 5-FU levels in patients with colorectal cancer undergoing continuous-infusion chemotherapy [2]. These observations support the rationale for therapeutic drug monitoring (TDM) during continuous-infusion 5-FU treatment.

Beyond BSA, fluorouracil disposition is influenced by hepatic catabolism through dihydropyrimidine dehydrogenase (DPD), circadian variation, age, sex, organ function, and regimen-specific factors [4,5]. Pretreatment pharmacogenetic screening for clinically relevant DPYD variants is increasingly recommended because it can identify patients at particularly high risk of severe early fluoropyrimidine toxicity [19,20]. However, genotype alone does not quantify the exposure achieved during a specific treatment cycle; accordingly, plasma-based monitoring remains relevant even in an era of genotype-informed supportive care.

Furthermore, the clinical implementation of TDM has historically been hampered by the “turnaround time gap”—the delay between sampling and receiving results often exceeds the infusion duration, rendering real-time dose adjustment impossible. A rapid, bedside monitoring system could bridge this gap, allowing clinicians to make informed decisions before the completion of a 48-h infusion, thereby maximizing the therapeutic index within a single cycle.

Implementation of 5-FU TDM in routine oncology practice has nevertheless remained limited. Barriers include the need for timely blood sampling, the complexity of chromatographic assays, and uncertainty regarding how a measured concentration should be translated into a clinically actionable dose adjustment [5,21,22,23,24,25,26,27]. Previous work on limited-sampling approaches, validated immunoassays, and practical dosing algorithms has substantially lowered these barriers [21,22,23,24,25,26,27]. These developments are particularly relevant if simplified monitoring tools can make TDM feasible in institutions without immediate access to specialized analytical equipment.

The conventional pharmacokinetic approach to 5-FU dose adjustment is based on the area under the concentration–time curve (AUC) [8,11,12,13]. Based on prior studies on pharmacokinetic-guided dosing and TDM [14,15,17,18,28,29], international recommendations published in 2019 proposed a target AUC range of 20–30 mg·h/L for 5-FU therapy [6]. Achieving exposure within this range may help optimize efficacy while limiting excessive toxicity.

In the present study, we examined the relationship between plasma 5-FU exposure and clinical outcome in patients with CRC, stratified by whether the target AUC range was achieved. We also evaluated a simple immunochromatography-based plasma 5-FU measurement kit developed to facilitate practical dose adjustment in routine clinical settings.

## 2. Materials and Methods

### 2.1. Study Design and Patients

This prospective, observational pilot study enrolled 15 patients with unresectable, advanced, or recurrent colorectal cancer who received continuous-infusion 5-fluorouracil-based chemotherapy at Tobu Chiiki Hospital (Tokyo, Japan) between 1 January 2017 and 30 April 2018. Eligibility criteria included histologically confirmed colorectal adenocarcinoma, planned treatment with a continuous-infusion 5-fluorouracil-containing regimen, and availability of serial plasma samples for pharmacokinetic evaluation. Clinical data, tumor response, adverse events, and survival outcomes were collected prospectively. The sample size was not based on a formal power calculation; rather, it reflected the number of eligible patients with serial pharmacokinetic data available during the study period. Accordingly, this study should be interpreted as exploratory and hypothesis-generating.

Performance status was evaluated using the Eastern Cooperative Oncology Group (ECOG) classification, and patients with ECOG performance status (PS) ≤ 2 were eligible [30]. All patients were treated under the supervision of professional medical staff. Patients were hospitalized for 4 days for each treatment cycle and were readmitted every 2 weeks.

### 2.2. Ethics

The study was conducted in accordance with the Declaration of Helsinki and was approved by the ethics committee of Tobu Chiiki Hospital (approved on 21 December 2015; IRB number 15.12.21 No. 4). Written informed consent was obtained from all participants.

### 2.3. Measurement of Plasma 5-FU Levels

**Figure 1:**
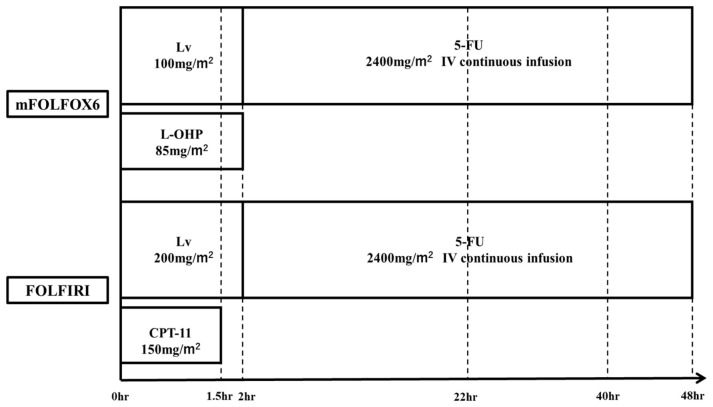
Treatment regimens and blood-sampling schedule. Dotted vertical lines indicate scheduled blood sampling before infusion (0 h) and at 22 h and 40 h after initiation of the continuous 5-fluorouracil infusion. Abbreviations: LV, leucovorin; L-OHP, oxaliplatin; CPT-11, irinotecan; BSA, body surface area.

Peripheral venous blood samples were collected during eight treatment cycles for each patient. In each cycle, samples were obtained at three time points: before the start of infusion and at 22 h and 40 h after initiation of the continuous 5-fluorouracil infusion. (Figure 1).

Plasma 5-fluorouracil levels were measured using the My-5FU^®^ competitive homogeneous nanoparticle agglutination immunoassay (FALCO Biosystems Ltd., Kyoto City, Japan). Because previous 5-FU TDM studies have reported substantial variability in systemic exposure and have used pharmacokinetic follow-up to support individualized dosing [5,6,18,29], we derived a patient-level descriptive exposure index from repeated measurements across cycles. Representative concentration values for each patient were defined as the median of the measured concentrations obtained over eight cycles. The median was used rather than the mean to reduce the influence of transient outliers and cycle-specific fluctuations in plasma 5-FU concentrations. The representative AUC was then calculated from these median concentrations. This approach was intended to describe each patient’s typical systemic exposure during repeated continuous-infusion therapy. It was not intended to replace formal cycle-specific pharmacokinetic analysis or real-time dose-adjustment monitoring.

**Figure 2:**
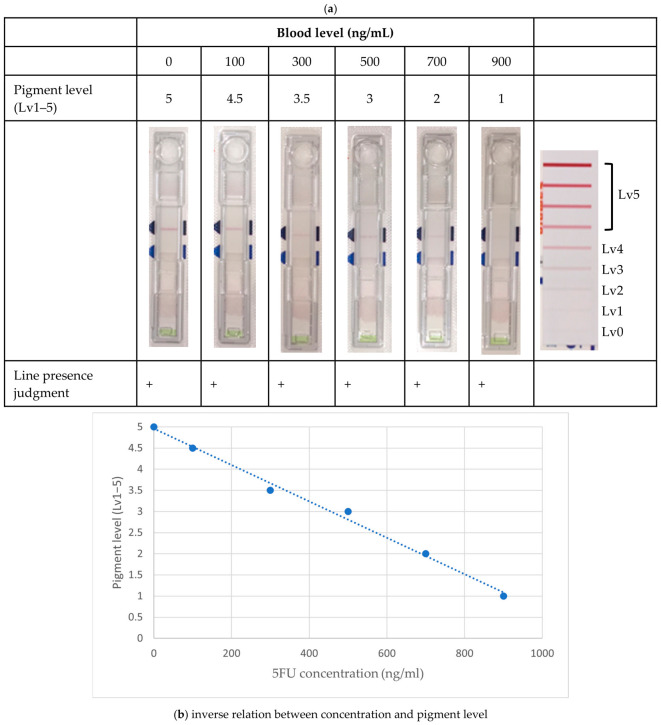
Prototype immunochromatographic assay and semi-quantitative signal readout. Representative strip appearance across increasing 5-FU concentrations (**a**) and the inverse relationship between 5-FU concentration and pigment level (**b**). Concentration levels are shown together with the corresponding pigment levels. The plot includes *X* and *Y* axes, the regression equation, and the R2 value. The color intensity of the signal line represents the semi-quantitative pigment level and is inversely related to the 5-FU concentration; the colors do not indicate different experimental groups.

As an alternative to the My-5FU assay, we developed a prototype plasma 5-FU measurement kit based on immunochromatography (IC), which can be used with either serum or plasma specimens. This prototype was developed as an in-house assay under development and was not a commercially validated product. Compared with laboratory-based analytical techniques such as LC-MS or HPLC, the IC-based kit yields measurements in approximately 10 min. Readout with an IC reader provides numerical values that approximate the target AUC range in plasma level monitoring (Figure 2). However, formal analytical validation, including limit of detection, limit of quantification, accuracy, precision, reproducibility, and inter-lot variability, remains necessary before clinical implementation.

In the present study, nine available paired samples were analyzed using both the prototype IC method and the My-5FU assay to perform a preliminary method-comparison assessment. Bland–Altman analysis was also performed to evaluate agreement between the two methods.

### 2.4. Clinical Evaluation and Survival Follow-Up

Adverse events during chemotherapy were evaluated according to the Common Terminology Criteria for Adverse Events (CTCAE), version 5.0, using findings from physical examination and laboratory testing. Toxicity severity was graded on a scale of 1 to 5, and adverse events were assessed on the last day before discharge for each treatment cycle.

Tumor response was evaluated after the 3-month monitoring period according to the Response Evaluation Criteria in Solid Tumors (RECIST) version 1.1 [31]. Complete response was defined as the disappearance of all target lesions; partial response as a reduction of more than 30% in the sum of lesion diameters; stable disease as insufficient shrinkage for partial response and insufficient increase for progressive disease; and progressive disease as an increase of more than 25% in at least one lesion or the appearance of new lesions.

Progression-free survival (PFS) and overall survival (OS) were assessed exploratorily using the available follow-up data. PFS was defined as the interval from treatment initiation to documented disease progression or death from any cause, whichever occurred first. Patients without progression were censored at the date of the last tumor assessment. OS was defined as the interval from treatment initiation to death from any cause, and surviving patients were censored at the date of the last confirmed survival.

### 2.5. Statistical Analysis

Statistical analyses were performed using JMP version 19 software (SAS Institute, Cary, NC, USA). Baseline continuous variables are presented as means with ranges, pharmacokinetic variables are presented as medians with ranges, and categorical variables are presented as numbers and percentages. Patients were grouped according to whether the representative 5-fluorouracil AUC fell within the predefined target range of 20–30 mg·h/L. Between-group comparisons of categorical variables were performed using Fisher’s exact test. Kaplan–Meier curves were generated for progression-free survival and overall survival, and exploratory log-rank *p*-values were calculated. A Bland–Altman analysis was performed to compare the prototype IC method with the My-5FU assay. Because of the small sample size and limited number of events, multivariable modeling and meaningful adjusted subgroup analyses were not performed to avoid overfitting. All *p*-values were two-sided, and *p* < 0.05 was considered statistically significant for exploratory analyses.

## 3. Results

The patient sample consisted of 15 patients, predominantly men (10/15, 66.7%). The mean age was 69 years, and the mean body surface area was 1.57 m^2^. ECOG performance status was 0, 1, and 2 in 1, 11, and 3 patients, respectively. Tumor location was the descending colon in 1 patient, the sigmoid colon in 9 patients, and the rectum in 5 patients. Clinical stage III disease was present in 1 patient, whereas 14 patients had stage IV disease. Eleven patients had undergone surgery, and 14 patients had metastatic disease. First-line chemotherapy regimens were heterogeneous and are summarized in Table 1.

The baseline characteristics of our cohort reflected a high disease burden, with 93.3% of patients presenting with Stage IV disease and 73.3% having undergone prior surgical resection of the primary tumor. First-line chemotherapy regimens were variable; the most common regimen was BV + FOLFOX (6 patients), followed by FOLFIRI (2 patients) and BV + FOLFOXIRI (2 patients). Other regimens were used in one patient each. This heterogeneity was taken into account when interpreting the exploratory association between the representative AUC and clinical outcomes.

The median representative AUC was 24.3 mg·h/L (range, 11.9–40.8). Eight patients (53.3%) were within the predefined target range of 20–30 mg·h/L, whereas seven patients (46.7%) were outside the range (Table 2).

Tumor control tended to be more favorable in patients within the target range. Specifically, 7 of 8 patients (87.5%) within the target range achieved either a partial response or stable disease, whereas 3 of 7 patients (42.9%) outside the target range showed disease control (Table 3).

Grade ≥2 adverse events associated with 5-fluorouracil treatment were observed in 2 of 8 patients (25.0%) within the target range and in 6 of 7 patients (85.7%) outside the target range, indicating a significant difference between the groups (*p* = 0.041) (Table 4).

To further explore the relationship between representative AUC and specific adverse events, we summarized Grade ≥ 2 adverse events within representative AUC subgroups as an exploratory supplementary analysis (Supplementary Table S1). Overall adverse-event severity according to representative AUC subgroup is summarized in Supplementary Table S2. Any Grade ≥ 2 adverse event was observed in 2 of 3 patients (66.7%) in the underexposure group, 2 of 8 patients (25.0%) in the target-range group, and 4 of 4 patients (100.0%) in the overexposure group. Several clinically relevant adverse events, including fatigue, lymphedema, diarrhea, stomatitis, anorexia, peripheral neuropathy, leukopenia, and neutropenia, appeared more frequently in the overexposure subgroup. However, because of the small number of patients and events, this analysis was descriptive and should be interpreted cautiously.

**Figure 3:**
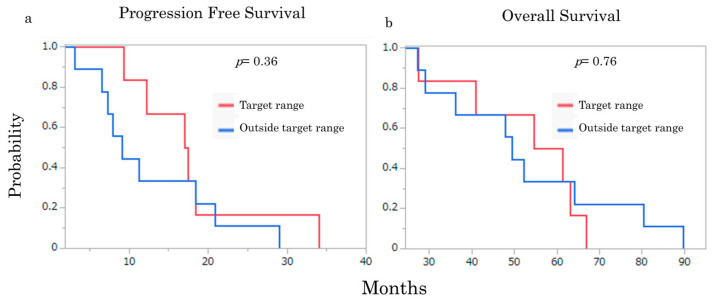
Kaplan–Meier curves for survival outcomes. (**a**) Progression-free survival. (**b**) Overall survival. Red line, target AUC range; blue line, outside target AUC range. *p*-values shown on the panels are exploratory log-rank *p*-values.

Kaplan–Meier analysis showed numerically longer progression-free survival in patients whose representative plasma 5-fluorouracil exposure was within the target range than in those outside the target range (17.2 months vs. 9.2 months, *p* = 0.36). Overall survival did not clearly differ between the two groups (*p* = 0.76). Because these differences did not reach statistical significance and the cohort was small, the survival analyses should be interpreted strictly as exploratory and hypothesis-generating (Figure 3).

**Figure 4:**
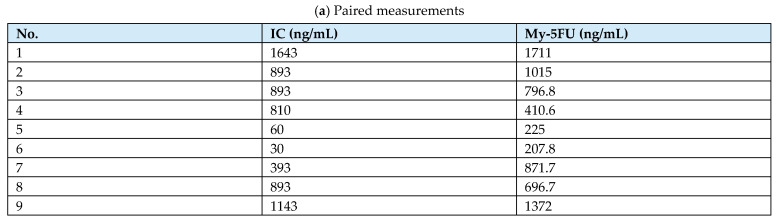

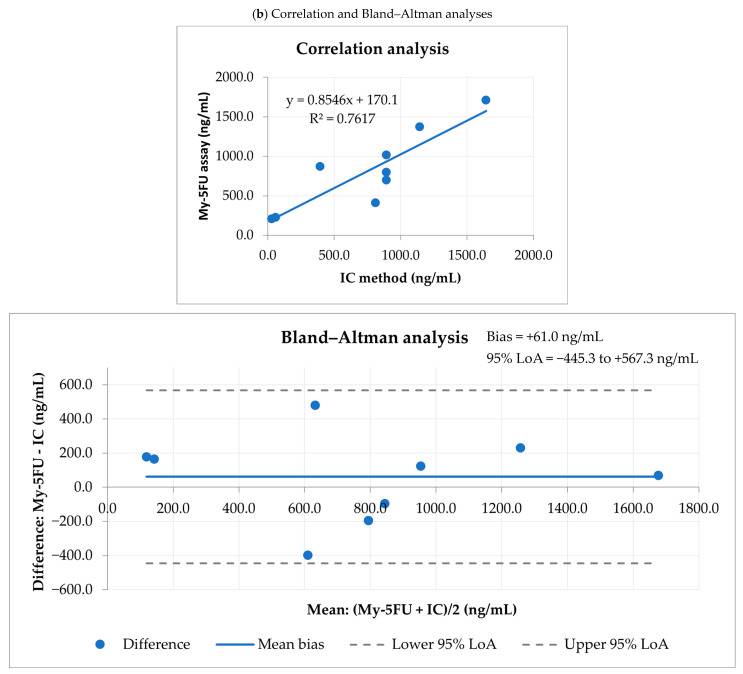
Method comparison between the immunochromatographic method and the My-5FU assay. (**a**) Correlation analysis between the IC method and the My-5FU assay measurements. The solid line indicates the linear regression line. (**b**) Bland–Altman analysis showing the difference between the two methods plotted against their mean. The difference was calculated as My-5FU minus IC. The solid line indicates the mean bias (+61.0 ng/mL), and the dashed lines indicate the 95% limits of agreement (−445.3 to +567.3 ng/mL). IC, immunochromatographic; LoA, limits of agreement. The colors in the plots are used only to distinguish data points and reference lines and do not indicate different patient groups.

We also evaluated the feasibility of the prototype IC method as a preliminary method-comparison assessment. In nine paired samples, the IC method showed a favorable correlation with the My-5FU assay (R2 = 0.762; Figure 4a). A Bland–Altman analysis was performed to evaluate the agreement between the two methods further. The mean bias, calculated as My-5FU minus IC, was +61.0 ng/mL, and the 95% limits of agreement ranged from −445.3 to +567.3 ng/mL (Figure 4b). Although the overall correlation was favorable, the limits of agreement were relatively wide, suggesting that this assay comparison should be considered preliminary.

Taken together, this preliminary method-comparison analysis indicates that the prototype IC method may be useful for rapid screening of plasma 5-FU levels, but the relatively wide limits of agreement and the small number of paired samples indicate that further analytical validation is required before it can be used for clinical dose adjustment.

## 4. Discussion

Multiple studies from Western countries have demonstrated marked inter-individual variability in the pharmacokinetics of 5-FU in patients with colorectal cancer and other malignancies, and this variability has been linked to both toxicity and therapeutic efficacy [8,9,10,11,12,13,14,15,16,17,18]. Previous Japanese studies have also suggested that higher plasma 5-FU levels are associated with a greater risk of adverse events [1]. In our previous preliminary study, we reported the feasibility of measuring plasma 5-FU levels in colorectal cancer patients receiving continuous-infusion chemotherapy [2]. In the present pilot cohort, patients whose representative plasma exposure fell within the predefined target AUC range had less frequent clinically relevant toxicity and, descriptively, more favorable disease control than those outside the range. However, these observations should be interpreted as associations rather than evidence of causality.

The treatment regimens were not uniform, and the cohort included patients who received different fluoropyrimidine-based combination regimens with or without molecularly targeted agents. This heterogeneity, together with differences in metastatic burden and prior surgical history, may have confounded the observed associations between representative AUC and clinical outcomes. Because of the small sample size and limited number of events, multivariable adjustment or meaningful stratified analysis was not feasible. Therefore, the observed associations should be interpreted as exploratory and hypothesis-generating.

Previous therapeutic drug monitoring studies have shown that pharmacokinetically guided dose adjustment of 5-FU can improve the therapeutic index [8,11,12,13]. Earlier reports established a target AUC range of approximately 20–24 mg·h/L in fluorouracil-based regimens [14,15,17,18,28,29], while later guidance broadened the recommended target range to 20–30 mg·h/L [6]. In Japanese patients with metastatic colorectal cancer, the a-JUST phase II trial of pharmacokinetic dose adjustment during modified FOLFOX7 plus bevacizumab also supported the feasibility of AUC-guided management [3]. In the present study, the median AUC was 24.3 mg·h/L, which fell within this clinically relevant range. This finding suggests that the target range derived from earlier studies may also be meaningful in our cohort.

Our results further suggest that achieving an appropriate systemic exposure to 5-FU may be relevant not only to toxicity management but also to treatment effectiveness. Disease control was more frequent in the target-range group, and grade ≥2 adverse events were less frequent in this group. Exploratory survival analysis showed numerically longer PFS in patients within the target range, whereas OS curves were broadly similar between the groups. Taken together, these findings are directionally consistent with previous reports indicating that pharmacokinetically guided treatment may improve response while reducing excessive toxicity and, in some cohorts, may contribute to favorable survival outcomes [8,18,28,29]. This interpretation is also concordant with more recent evidence. In a comparative study of colorectal cancer patients who received fluorouracil-based chemotherapy, TDM was associated with a lower rate of severe adverse events and improved efficacy relative to historical controls [32]. In addition, a meta-analysis of prospective controlled studies showed that pharmacokinetic-guided 5-FU dosing improved objective response rates and reduced grade 3/4 adverse events compared with BSA-based dosing [7].

An additional contemporary issue is how plasma-based TDM should be integrated with pretreatment DPD assessment. Prospective data indicate that DPYD genotype-guided dose reduction can improve the safety of fluoropyrimidine treatment in variant carriers [19], and CPIC recommendations provide a practical framework for genotype-informed starting doses [20]. Swiss expert recommendations have further emphasized that DPYD genotyping and TDM should be regarded as complementary rather than competing strategies for fluoropyrimidine individualization [33]. At the same time, pretreatment genotyping does not replace direct measurement of drug exposure during treatment. In practice, pharmacogenetic screening and TDM may address different components of risk: the former identifies patients predisposed to marked toxicity, whereas the latter verifies whether the delivered regimen achieves an acceptable exposure window in the individual patient. This complementary framework may become particularly important if simplified assays make real-time exposure assessment more accessible [34]. 

A notable feature of the present study is that plasma 5-fluorouracil exposure was evaluated longitudinally across eight treatment cycles. The use of median concentrations across cycles to derive a representative AUC was intended to reduce the influence of transient outliers and provide a stable patient-level estimate of typical exposure. Nevertheless, this median-based representative AUC is an exploratory methodological approach and has not been formally validated as a cycle-specific dose-adjustment endpoint. It may mask clinically relevant intra-patient variability, including transient high-exposure cycles associated with toxicity or low-exposure cycles associated with reduced efficacy. Therefore, representative AUC should be interpreted as a descriptive patient-level exposure index. Future studies should evaluate cycle-specific AUC values, intra-patient variability, and the temporal relationship between exposure, adverse events, and tumor response.

Given the relationship between target AUC attainment and adverse events observed in the present study, dose adjustment based solely on body surface area may be insufficient to optimize 5-fluorouracil treatment. In clinical practice, a TDM-based dose-adjustment framework would compare the measured 5-FU AUC with the target range of 20–30 mg·h/L. Underexposure without clinically significant toxicity may support cautious dose escalation in subsequent cycles, whereas overexposure or clinically significant toxicity may support dose reduction, treatment delay, or intensified supportive care. Patients within the target range may generally continue the same dose if toxicity and disease control are acceptable. However, this approach should be used as clinical decision support rather than as an automatic dosing rule, and decisions should also consider regimen, toxicity profile, organ function, performance status, and tumor response. Because this study did not prospectively test AUC-guided dose modification, this framework requires validation in future studies.

DPYD genotyping and DPD deficiency screening were not performed in this cohort. This is an important limitation because fluoropyrimidine metabolism varies substantially among patients, and even a single patient with markedly impaired DPD activity could influence exposure and toxicity findings in a small cohort. Future studies should integrate pharmacogenetic screening with plasma-based TDM to evaluate complementary strategies for fluoropyrimidine individualization.

The prototype IC assay also requires cautious interpretation. Correlation analysis showed a favorable association between the IC method and the My-5FU assay, but Bland–Altman analysis demonstrated relatively wide limits of agreement. Therefore, the present comparison should be regarded as a preliminary method-comparison and feasibility assessment rather than a full analytical validation. Larger studies should assess accuracy, precision, reproducibility, limit of detection, limit of quantification, and inter-lot variability before the assay is used for clinical dose adjustment.

In the exploratory supplementary analysis of specific adverse events, Grade ≥2 adverse events appeared more frequent in the overexposure subgroup than in the target-range subgroup. This observation is biologically plausible because excessive systemic 5-FU exposure may increase fluoropyrimidine-related toxicities, including mucosal injury, gastrointestinal toxicity, myelosuppression, and hand-foot syndrome. However, the number of patients in each subgroup was small, and multiple adverse-event categories could occur in the same patient. Therefore, this analysis cannot establish toxicity-specific mechanisms, and the findings should be regarded as exploratory and hypothesis-generating.

At the same time, the findings related to tumor control and survival should be interpreted cautiously. Because of the small sample size, single-center design, treatment regimen heterogeneity, lack of multivariable adjustment, lack of DPYD data, and exploratory follow-up analysis, our data do not allow us to conclude that achieving the target AUC independently improved progression-free survival or overall survival. Rather, these observations should be regarded as hypothesis-generating and require confirmation in larger prospective studies.

## 5. Conclusions

In conclusion, target plasma 5-fluorouracil exposure was associated with lower clinically relevant toxicity and may support favorable tumor control in this pilot cohort. The prototype immunochromatographic method demonstrated preliminary feasibility for rapid plasma 5-FU monitoring, although further validation is required before routine dose adjustment.

## Figures and Tables

**Table 1:** Patient characteristics.

Characteristic	Value
Age, years	69 (52–79)
Male/Female	10/5
BSA, m^2^	1.57
ECOG PS, 0/1/2	1/11/3
Tumor location, descending/sigmoid/rectum	1/9/5
Pathological type, well/moderate/papillary	4/10/1
Primary/Recurrent	9/6
Clinical stage, III/IV	1/14
Metastasis, yes/no	14/1
Location of metastasis, liver/lung/peritoneum/multiple	6/1/2/5
Operation, yes/no	11/4
First-line chemotherapy regimen, *n*	
BV + FOLFOX	6
FOLFIRI	2
BV + FOLFOXIRI	2
CET + mFOLFOX	1
CET + mFOLFIRI	1
FOLFOX	1
Pmab + FOLFIRI	1
BV + FOLFIRI	1

Age is shown as mean (range); BSA is shown as mean. BV, bevacizumab; CET, cetuximab; Pmab, panitumumab; FOLFOXIRI, fluorouracil, leucovorin, oxaliplatin, and irinotecan.

**Table 2:** Plasma 5-FU AUC by sampling time and target-range status.

Collection Time	Target AUC Range (20–30 mg·h/L) (*n* = 8)	Outside Target Range (*n* = 7)
0 h	0	0
22 h	22.1 (20.6–26.8)	17.2 (10.1–36.5)
40 h	25.5 (21.6–28.4)	18.1 (11.9–40.8)

Values are median (range). AUC, area under the concentration–time curve.

**Table 3:** Best tumor response according to target-range status.

Best Response	Target AUC Range (20–30 mg·h/L) (*n* = 8)	Outside Target Range (*n* = 7)
PR or SD	7 (87.5%)	3 (42.9%)
PD	1 (12.5%)	4 (57.1%)

PR, partial response; SD, stable disease; PD, progressive disease. Fisher’s exact test for disease control (PR/SD vs. PD): *p* = 0.119.

**Table 4:** Toxicity according to target-range status and serial AUC among patients with grade ≥ 2 toxicity. (**a**) Incidence of Grade ≥ 2 Toxicity. (**b**) Plasma AUC Among Patients with Grade ≥ 2 Toxicity.

(**a**)		
**Toxicity Grade**	**Target AUC Range** **(20–30 mg·h/L)** **(*n* = 8)**	**Outside Target Range** **(*n* = 7)**
<2	6 (75.0%)	1 (14.3%)
≥2	2 (25.0%)	6 (85.7%)
(**b**)		
**Group**	**22 h AUC (mg·h/L)**	**40 h AUC (mg·h/L)**
Target AUC range (20–30 mg·h/L) (*n* = 2)	23.6 (21.5–26.8)	25.3 (23.3–28.4)
Outside target range (*n* = 6)	17.6 (10.3–36.5)	18.9 (11.9–40.8)

Values in panel (b) are median (range). AUC, area under the concentration–time curve.

## Data Availability

The data presented in this study are available from the corresponding author upon reasonable request. The data are not publicly available because of privacy and institutional restrictions.

## Supplementary Materials

The following supporting information can be downloaded at: https://www.mdpi.com/article/10.3390/cancers18101673/s1. Table S1: Specific Grade ≥ 2 adverse events according to representative AUC subgroup; Table S2: Overall adverse-event severity according to representative AUC subgroup.

## Abbreviations

The following abbreviations are used in this manuscript:

5-FU	5-fluorouracil
AUC	area under the concentration–time curve
BSA	body surface area
CRC	colorectal cancer
CTCAE	Common Terminology Criteria for Adverse Events
ECOG	Eastern Cooperative Oncology Group
IC	immunochromatography
mFOLFOX6	modified FOLFOX6
OS	overall survival
PFS	progression-free survival
PS	performance status
RECIST	Response Evaluation Criteria in Solid Tumors
TDM	therapeutic drug monitoring
